# Late Water Deficits Improve Intrinsic Water Use Efficiency, Fruit Maturity, and Acceptability in Yellow-Fleshed Kiwifruit cv. Soreli

**DOI:** 10.3390/plants14182843

**Published:** 2025-09-12

**Authors:** Arturo Calderón-Orellana, Mauricio Calderón-Orellana, Catalina Atenas, Carolina Contreras, Felipe Aburto, Tamara Alvear, Silvia Antileo-Mellado

**Affiliations:** 1 Departamento de Producción Vegetal, Facultad de Agronomía, Universidad de Concepción, Chillán 3780000, Chile; talvear2019@udec.cl (T.A.); santileo2016@udec.cl (S.A.-M.); 2 Centro de Evaluación Rosario, Rengo 2940000, Chile; mcalderon@olivos.cl (M.C.-O.); catenas@cerosario.cl (C.A.); 3 Instituto de Producción y Sanidad Vegetal, Facultad de Ciencias Agrarias y Alimentarias, Universidad Austral de Chile, Valdivia 5091000, Chile; carolina.contreras@uach.cl; 4 Soil and Crop Sciences Department, College of Agriculture and Life Sciences, Texas A&M University, 370 Olsen Blvd., TAMU 2474, College Station, TX 77845, USA; felipe.aburto@tamu.edu

**Keywords:** deficit irrigation, *Actinidia chinensis*, water use efficiency, fruit quality, sensorial attributes, hydric stress

## Abstract

Water scarcity poses a significant threat to kiwifruit production, especially in Mediterranean climates. This study investigated the impact of late-season regulated deficit irrigation (RDI) on water use efficiency and fruit quality of yellow-fleshed kiwifruit (*Actinidia chinensis* cv. Soreli) over two seasons in central Chile. Four irrigation treatments were applied during fruit ripening: full irrigation (Control), moderate deficits for three or five weeks (D50S and D50L), and complete irrigation suspension for three weeks (D100). While D100 had minimal impact on stomatal conductance, it significantly reduced stem and leaf water potentials, indicating severe water stress. D100 treatment also showed the highest intrinsic water use efficiency (via δ13C enrichment) and improved water productivity by up to 20%. Fruits from D100 and D50S had higher soluble solids (up to 2.0 °Brix) without compromising firmness or yield. Sensory evaluations indicated greater consumer acceptance in water-stressed treatments, especially D100, due to enhanced color and flavor. Principal component analysis confirmed that moderate-to-severe-water stress correlated with favorable sensory profiles. These findings suggest that short-term, late-season water deficits can enhance fruit quality and water use efficiency without reducing yield, offering a sustainable strategy for kiwifruit production under increasing water limitations.

## 1. Introduction

Chile is one of the leading producers of kiwifruit, with approximately 7500 hectares cultivated [1]. However, Chile’s central regions, the main producers of kiwifruit, have experienced a substantial reduction in irrigation water due to a progressive decline in winter rainfall over the last decade [2]. This is consistent with the patterns of the ongoing megadrought, which has led to a 20–30% reduction in annual rainfall over the last decade in these areas [3].

Under the worst-case scenario, climate change models predict that central regions of Chile may experience a significant decrease in snow water and a substantial increase in mean air temperature (−39% and +4.1 °C, respectively) [4]. This situation may generate unprecedented consequences for Chilean agriculture, especially for fruit crops like kiwifruit, as they require considerable amounts of irrigation water (~10,000 m^3^ ha^−1^) to produce high marketable yields (>50 tons ha^−1^) [5]. Kiwifruit plants (*Actinidia* spp.) are highly sensitive to low water availability due to their shallow root systems and high transpiration rates [6]. Water stress often increases the concentration of abscisic acid (ABA) and induces stomatal closure in many fruit crops, but kiwifruit vines generally exhibit a weak stomatal response to soil water depletion [7]. This weak response clearly results in low tolerance to water stress and high vulnerability to xylem embolism [8]. However, when kiwifruit plants are subjected to severe water stress (midday SWP < −1.3 MPa), stomatal closure can be high enough to significantly reduce photosynthetic rates and leaf area, reaching up to 64% and 76% reduction, respectively [9]. If kiwifruit plants reach severe water stress levels near flowering, considerable reductions in yield (~25%) may be expected [10].

In response to changes in water availability for irrigation, Chilean fruit crops have progressively migrated from more arid zones to southern and wetter regions of the country [11]. However, this strategy does not effectively address the urgent need to implement water conservation practices in areas currently dedicated to kiwi cultivation, especially considering that the central part of the country accounts for more than 90% of the established area of this fruit crop [12]. In this context, regulated deficit irrigation (RDI) is one of the most water-conservative irrigation practices used in fruit production. The amount of water applied in fruit orchards under RDI does not satisfy the whole crop evaporative demand for a limited period of the growing season, when reproductive growth and development is less sensitive to water stress [13]. In kiwifruit orchards, RDI must be applied several weeks before harvest to prevent fruit size and yield reductions [7]. In fact, moderate water stress during ripening increases soluble solids accumulation and reduces fruit softening at postharvest [10]. The impact of water stress on plant physiology, vegetative, and reproductive growth in kiwifruit varies among different cultivars. Pratima and Sharma [6] showed high variability in midday leaf water potentials, stomatal conductance, chlorophyll concentration, leaf area, shoot growth, and yield estimates among five green-fleshed kiwifruit cultivars.

Yellow-fleshed kiwi cultivars have gained increasing interest in international markets due to their high productivity and the expression of highly valued quality attributes, such as intense pulp coloration, low fibrousness, a well-balanced sugar-to-acid ratio, and sensory profiles that include citrus and tropical notes [14]. The demand for vitamin-rich fruits has increased significantly due to the impact of the Coronavirus disease (COVID-19) pandemic [15]. Consumption of yellow kiwifruit increased, as it contains twice the vitamin C of oranges. For instance, Zespri reported record sales for yellow-fleshed kiwifruits, with exports exceeding 200 million trays globally by 2023 [16]. In general, many of these cultivars are distinguished by their accelerated fruit ripening rates and more compact growing seasons compared to the green-fleshed ‘Hayward’ [17]. In Mediterranean regions, a shorter growing season can result in significant savings in seasonal water application. However, the rapid ripening often coincides with periods of elevated air temperatures and peak crop evaporative demand, which may compromise fruit quality. Therefore, evaluating irrigation strategies across multiple seasons is essential to account for interannual climate variability and to determine the stability of physiological responses, yield, and fruit quality under diverse environmental scenarios [18].

In yellow-fleshed cultivars, RDI has shown the capacity to improve fruit quality traits, including increased soluble solids and enhanced firmness at both the harvest and postharvest stages [19]. However, Boini et al. [20] demonstrated that a mild level of water stress (midday stem water potential of −0.5 MPa) does not affect gas exchange parameters and fruit growth in *Actinidia deliciosa*, though it does impact *Actinidia chinensis*, leading to changes in final yield. These results highlight the pivotal role of validating RDI in various A. chinensis cultivars and under diverse environmental conditions to determine the appropriate irrigation practices. The objective of this study was to evaluate the effects of regulated deficit irrigation (RDI) at the end of the season on plant water relations, water use efficiency, fruit quality, and productivity of *Actinidia chinensis* cv. ‘Soreli’ during two consecutive seasons, in order to identify optimal irrigation practices under variable environmental conditions between seasons, particularly in water-limited Mediterranean climates.

## 2. Materials and Methods

### 2.1. Description of the Study Site and Weather Data

**Figure 1:**
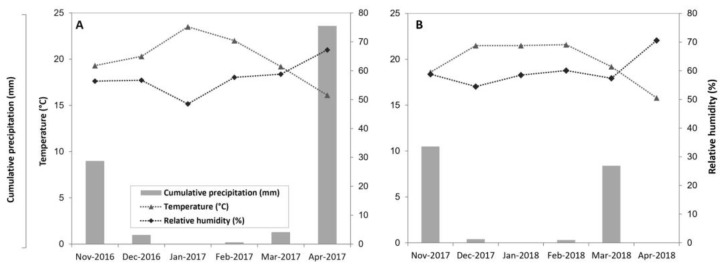
Monthly mean temperature (°C) and relative humidity (%) and cumulative precipitation (mm) from the San Fernando weather station (Red FDF-Agromet: 34°35′ S; 70°56′ W) in (**A**) 2017 and (**B**) 2018.

A field study was carried out in a seven-year-old kiwifruit orchard (*Actinidia chinensis* Chev. cv. Soreli) grafted onto ‘Bruno’ seedling rootstock in San Fernando (O’Higgins Region, Chile) (34°34′ S, 70°56′ W) for two consecutive growing seasons (2017 to 2018). Kiwifruit vines were drip irrigated (4 L h^−1^ emitters, spaced every 0.89 m along the row), planted at 3.5 m × 2.0 m spacing, trained as a pergola system with four cordons with ten to twelve eight-bud canes per cordon. Soils in the study site have been locally described as members of the Talcarehue soil series, and are classified as a coarse loamy calcareous mix, thermic Fluventic Xerochrepts [21]. Reference evapotranspiration (ET_o_) and daily and monthly climate data were obtained from the San Fernando weather station (FDF-Agromet Network: 34°35′ S; 70°56′ W), located approximately 10 km from the study site (see Figure 1). Daily estimates of crop evapotranspiration (ET_c_) were obtained as the product of ET_o_ and the crop coefficient (kc) for kiwifruit (FAO). Phenological stages were recorded when 50% of plants exhibited characteristic stage features according to the BBCH scale for kiwifruit [22]. Before bloom, lateral flower buds were manually thinned to increase fruit size at harvest, while fruit thinning was carried out after fruit set to eliminate deformed fruits. Pest and weed management were conducted according to standard commercial practices for kiwifruit orchards.

### 2.2. Irrigation Treatments and Experimental Design

Four irrigation treatments were applied at several periods during fruit maturity, following a randomized complete block design with repeated measurements over two consecutive seasons (2017 and 2018) in each irrigation plot. Each treatment was replicated four times in 0.5 ha plots arranged from south to north. The experimental unit consisted of 15 plants per block–treatment combination, distributed across three adjacent rows, with measurements taken from the central row to minimize border effects. The irrigation treatments were as follows: (i) Control plants (Control) were irrigated at 100% ET_c_ throughout the whole growing season; (ii) Long-period moderate water deficit (D50L): plants were irrigated as the Control from budbreak to five weeks before commercial harvest, and irrigation was thereafter reduced by 50% until fruit was collected; (iii) Short-period moderate water deficit (D50S): plants were irrigated as the Control from budbreak to three weeks before commercial harvest, and irrigation was thereafter reduced by 50% until fruit was collected; (iv) Short-period severe water deficit (D100): plants were irrigated as the Control from budbreak to three weeks before commercial harvest, and irrigation was thereafter completely ceased until fruit was collected. Water was applied in all irrigation treatments when midday stem water potential reached −1.3 MPa (see Supplementary Figure S1).

### 2.3. Environmental Conditions

Photosynthetic photon flux density (PPFD) was measured once a week above and below the canopy using a ceptometer (LP-80, Decagon Instruments, Washington, DC, USA) from 1 November to 30 March in both seasons. Data collection was carried out at solar noon, with one measurement taken 0.5 m above the upper canopy.

Temperature and relative humidity above the canopy were recorded from 1 November to 30 April in both seasons using an air temperature and humidity probe (HMP60, Vaisala, Helsinki, Finland) installed 0.5 m above the canopy in Control plants. Measurements were taken every 30 s, and data were logged every 5 min. These data were used to calculate air vapor pressure deficit (VPDₐᵢᵣ) using the following equation [23].

(1) $${\mathrm{VPD}}_{\mathrm{air}}=0.6108\;\cdot \;\exp\;\left(\frac{17.27\;\cdot \;T}{T+237.3\;}\right)\;\cdot \;1-\left(\frac{\mathrm{RH}}{100}\right)$$

where RH is air relative humidity and T is air temperature.

### 2.4. Plant and Soil Water Status

Leaf and stem water potential values were measured weekly at midday (12:00 to 15:00 h) under cloud-free conditions using a pressure chamber (Model 615, PMS Instruments, Corvallis, OR, USA), following the protocol described by McCutchan and Shackel [24]. Measurements were taken from two leaf samples per plant.

Stomatal conductance was estimated using a steady-state porometer (SC-1, Decagon Devices, Pullman, WA, USA) on the same plants where leaf water potential was measured. Two leaves per plant were evaluated using automatic mode to minimize sampling time.

Volumetric soil water content was monitored daily throughout the growing season for each treatment combination using dielectric sensors (GS1, Decagon Devices, Pullman, WA, USA) installed between irrigation emitters at depths of 30 and 60 cm. These sensors were connected to data loggers (EM50, Decagon Devices, Pullman, WA, USA), which recorded data every 10 min. The collected data were transmitted to the laboratory and processed to calculate the available water in the soil, expressed as the mean of both depths in percent.

### 2.5. Water Use Efficiency and Water Productivity

At harvest, a 10-leaf sample was randomly taken from the upper canopy in two vines from each experimental unit in order to estimate intrinsic water use efficiency, measured as differences in carbon isotope ratio (δ13C). Sampled leaves were mature and fully developed in size. Once leaf samples were collected, they were dried at 70 °C in an oven until the sample weight was stable. Dried samples were ground and sieved to obtain a homogeneous fine powder. Stable carbon isotopic ratios (δ13C) were determined using an EA-GSL gas preparation module (Sercon, Crewe, UK) coupled to an Isotope Ratio Mass Spectrometer (20–22 IRMS, Sercon, Crewe, UK). An ultra-grade reference gas (Ultra High-Grade CO_2_, Linde Group, Dublin, Ireland) was injected before each analytical run for CO_2_ drift correction. A calibrated laboratory standard (Corn Flour SCC2256, Sercon, Crewe, UK) was run every ten analytical samples. A standard sample was checked every ten analytical samples as an internal check for analytical quality. The stable carbon isotope composition (δ13C) for each sample was determined with the following equation [25]:

(2) δ13C ‰=C13C12sample-C13C12standardC13C12standard · 1000

where ^13^C/^12^C_sample_ and ^13^C/^12^C_standard_ are the measured ^13^C/^12^C ratios for the leaf sample and the PDB standard (Pee Dee Belemnite), respectively.

Water productivity was estimated as the product of the ratio between water applied (irrigation + precipitation) from budbreak to harvest (m^3^ ha^−1^) and yield per hectare (kg ha^−1^). Volumetric water meters (Dishnon, Arad Ltd., Dalia, Israel) were installed at the beginning of each irrigation line in one block-treatment combination to measure the amount of irrigated water from 1 October to 30 April in 2017 and 2018.

### 2.6. Vegetative Growth and Microclimate

Canopy coverage during the season was estimated as intercepted PPFD at 50% flowering and harvest time with a portable ceptometer (LP-80, Decagon Devices, Pullman, WA, USA). Four PPFD measurements below the plant canopy (1.5 m above the soil surface) were made at the center of the row, right below the plant arm, the edge of the ridge (0.5 m from the trunk), and at the center of the inter-row space (2.0 m from the trunk). An outside PPFD reading was made at a height of 0.5 m above each canopy. The percentage of intercepted PPFD was calculated using the following equation:

(3) $$\mathrm{IPPFD}\;=\left(1-\frac{\mathrm{PPFDin}}{\mathrm{PPFDout}}\right)\cdot \;100$$

where IPPFD was the percentage of intercepted PPFD by the vine canopy, PPFD_in_ was the average value of the four readings below the kiwifruit vine, PPFD_out_ was the reading above the vine canopy. Canopy and fruit temperatures were measured in each plant, and leaf water potential was determined using an infrared portable thermometer (Fluke 62Max, Fluke Corporation, Berlin, Germany). Temperature readings for the canopy and fruits were taken at 100 and 5.0 cm from the sample, respectively. Those distances were adequate to measure 50 cm^2^ and 10 cm^2^, following the manufacturer’s instructions. Chlorophyll concentration in leaves was estimated using a portable chlorophyll meter (MC-100, Apogee Instruments, Logan, UT, USA) in ten randomly selected well-exposed leaves per block-treatment combination.

### 2.7. Fruit Maturity and Quality at Harvest and Postharvest

Maturity evaluations were carried out weekly from the onset of fruit ripening to harvest time in randomly taken samples of twelve fruits per experimental unit. Harvest time was defined when the concentration of soluble solids was 9.2% and flesh hue reached 100 (yellow). Soluble solids concentration (SSC) was evaluated with a hand-held digital refractometer (HI 96801, Hanna Instruments, Smithfield, RI, USA). The color of the peel and flesh (hue) was evaluated with a portable colorimeter (CR-10, Konica-Minolta, Tokyo, Japan). At harvest, 360 fruits per experimental unit were randomly collected from three contiguous plants and immediately transported to the laboratory for quality evaluations. The first subgroup of 60 fruits (F1) was separated to measure fruit quality 24 h after harvest. A second and third subgroup of 150 fruits each was separated to measure fruit quality after cold storage at 0 °C for 30 and 60 days. Fruit firmness was evaluated using a fruit texture analyzer (DA-Meter, TR Turoni, Forli, FC, Italy) with a 7.9 mm plunger. For this evaluation, the peel was removed at two extremes: the insertion place of the instrument, and the maximum force required to completely insert the probe in the fruit was recorded. Two measurements were made for fruit at the central sites on each opposing cheek of the fruit. The percentage of dry matter in cross-sections approximately 2 to 3 mm thick, obtained from the equatorial area of the fruits, was estimated. The samples were individually weighed with a precision balance and then taken to a dehydrator at 70 °C until they reached a constant weight. The samples were weighed again to obtain the difference in weight. This result was expressed as a percentage of dry sample weight. In the harvest, the total fruits were counted and weighed per plant, which obtained the average weight of fruit. The same procedure was performed on all treated plants.

### 2.8. Sensory Evaluations

A sensory evaluation was carried out to determine the most relevant fruit quality attributes picked by the consumer after the irrigation treatments. Sensory analyses were performed after 90 days of cold storage at 0 ± 1 °C [26] and lasted 5 days at 20 °C. A trained panel was composed of fifteen people, who were recruited each year for the training process. The recruitment of the panel consisted of passing a basic recognition test for the taste and color of kiwifruit parameters. The panel consisted of women aged 21–34 years old, and men 19–35 years old. The training was performed in three sessions of 1 h each, with all sessions and tests being conducted in the same laboratory under the same light and temperature conditions.

Each panelist was served 1 kiwifruit cut into 4 pieces on a plate labeled with three random digits. Only ripened kiwifruits of a range of firmness values of 2–3 lbs measured with a firmness texture analyzer (FTA) were used for the sensory tests. Each panelist was asked to rate kiwifruit quality attributes divided into three categories: (i) Color intensity of the peel, flesh, and visual defects associated with decay; (ii) Aroma intensity, typicity and off-odors that differ from the typical kiwifruit aroma; (iii) Flavor measured as sweetness, acidity, taste, astringency, texture, and overall acceptability. For texture, specific descriptors were defined such as ‘hardness’, cohesiveness’, chewiness’, juiciness’, crunchiness’, crispness’, and ‘melting’ according to Contador et al. [27].

### 2.9. Statistical Analysis

The data were subjected to an analysis of variance (ANOVA) after testing for normality errors (Shapiro–Wilk), homogeneity of variances (Levene’s test), and additivity of effects (Tukey). Differences between means were determined using the LSD test (alpha = 0.05). All statistical analyses were performed using the statistical software SAS 9.4 (SAS Studio, University Edition, SAS Institute, Cary, NC, USA). To explore the relationship between the sensory test results and the irrigation treatments after 60 days of storage, a principal component analysis (PCA) was performed using the available software R v.4.0.3 (R Development Core Team, 2008). The data for 2017 and 2018 was independently studied. The analysis was carried out on twenty variable loadings distributed in three categories: color, aroma, and taste. On the other hand, the sample scores were composed of the four irrigation treatments previously described. Data analysis and interpretation was carried out according to Quinn and Keough [28].

## 3. Results

### 3.1. Environmental Conditions

The highest seasonal PPFD values were exhibited during the summer months (December to February), with maximum values close to 2000 µmol m^−2^ s^−1^ in January for both years (Figure 1A). Thereafter, PPFD progressively declined from March to April, falling to approximately 50% of peak levels by April. Despite minor seasonal differences in November and March, the overall seasonal pattern remained consistent throughout both years.

**Figure 2:**
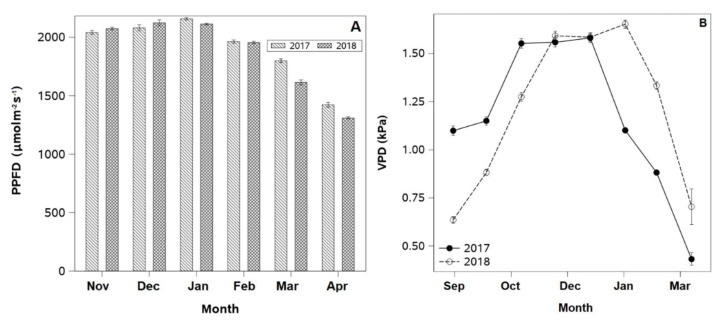
(**A**) Monthly average of photosynthetic photon flux density (PPFD) from anthesis to early senescence (November–April) and (**B**) Two-week average of air vapor pressure deficit (VPD) from budbreak to harvest (September to March) measured at 0.5 m above the plant canopy of ‘Soreli’ kiwifruit vines in 2017 and 2018. Error bars represent ±1 se.

Analysis of monthly VPD averages from September to April revealed greater seasonal variability than the PPFD pattern (Figure 2A,B). In the first year, peak VPD values were observed in October (approximately 1.5 kPa), followed by a gradual decline through the summer months (Figure 1B). Minimum values were reached in March (near 0.5 kPa). In the second year, maximum VPD values were reached in November, one month later than in the previous year. Additionally, the second season showed a more gradual and delayed decline in VPD, with VPD values nearly 45% higher between February and April.

### 3.2. Water Application, Yield, and Water Productivity

The amount of total water applied during the season varied from year to year (Table 1). In the second season, water application to the Control treatment was 9% lower than in the first season. The D100 treatment achieved the highest water savings for both years, reaching a 20% reduction in water application compared to the Control in the first season. In the second season, the difference between the treatments was less than 10%. The number of fruits per vine, counted after fruit set and at harvest, showed no difference among irrigation strategies each year. Yield per hectare was also similar among irrigation treatments, reaching approximately 26 tons ha^−1^ each season (Table 1). Each deficit irrigation treatment improved water productivity compared to the Control, ranging from 9% to 20% and from 4% to 12% in 2017 and 2018, respectively. However, the D50L treatment reached the highest water productivity in both years. (Table 1).

### 3.3. Plant and Soil Water Status

**Figure 3:**
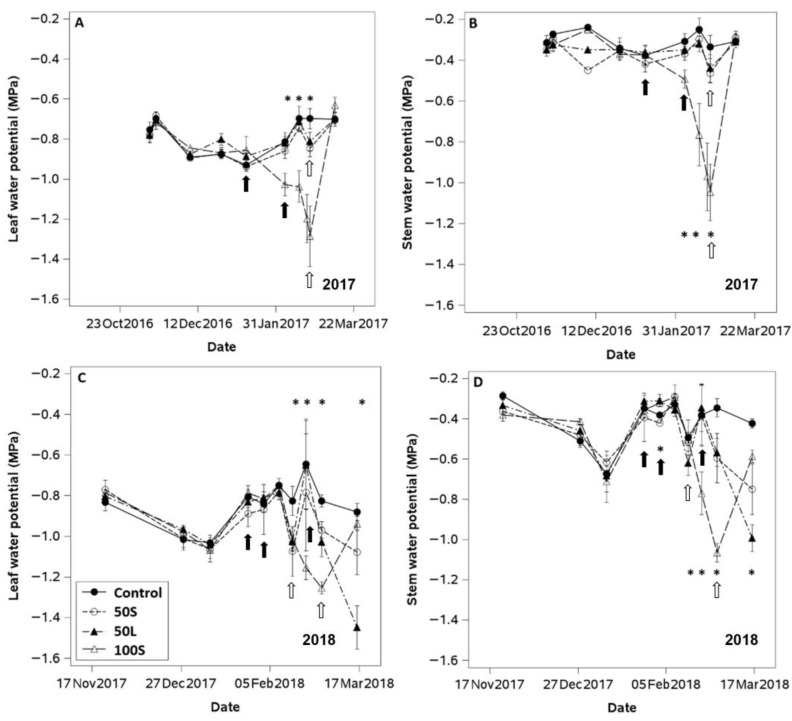
Average values of (**A**) midday leaf water potential, (**B**) midday stem water potential measured from anthesis to harvest time (November–5 March) in 2016–2017, and (**C**) midday leaf water potential and (**D**) midday stem water potential measured from anthesis to harvest time (November–17 March) in 2017–2018 in ‘Soreli’ kiwifruit vines subjected to four irrigation treatments (Control: 100% ET_c_, D50S: 50% ET_c_ for three weeks before harvest, D50L: 50% ET_c_ for five weeks before harvest, and D100: 0% ET_c_ for three weeks before harvest). Black arrows indicate water cuts in the treatments, and white arrows indicate irrigation replenishment. Asterisks indicate significant differences (*p* ≤ 0.05, n = 4) by LSD test. Error bars represent ±1 se.

At the beginning of each season, all irrigation treatments exhibited similar plant water status values, with stem and leaf water potentials around −0.35 and −0.75 MPa, respectively (see Figure 3A–D). Thereafter, the water status of all plants decreased slightly until deficit irrigation treatments were applied at the end of January in the first season and at the beginning of February in the second season. After irrigation treatments were applied, plants subjected to deficit irrigation showed significant reductions in stem and leaf water potential. However, in the first season, only D100 exhibited large differences in plant water status compared to the Control treatment. In the second season, the differences in plant water status among the irrigation treatments were larger than in the previous season.

The application of irrigation treatments in the second season coincided with an increase in leaf and stem water potentials in all plants as they recovered from an unexpected failure of the irrigation system at the beginning of January. In 2017, plants from the D100 and D50L treatments exhibited the lowest leaf and stem water potential values. However, these minimum values were reached at different times. D100 plants reached maximum water stress levels fifteen days before D50L plants. Plants from the D100 and D50L irrigation treatments showed similar minimum values of SWP (−1.3 MPa) at the end of the second season. However, D100 exhibited LWP values that were 0.2 MPa higher than those from the D50S.

Stomatal conductance was similar for all plants before irrigation treatments were applied at the end of January (Table 2). The values of stomatal conductance were the highest in December, reaching an average of 731 mmol m^−2^ s^−1^. Stomatal conductance then decreased progressively until the last measurement in March. At the end of the season, D100 plants showed the lowest stomatal conductance one week before D50L plants. However, there was a significant difference in the decrease in stomatal conductance, with D100 plants showing a 30% reduction compared to the 18% reduction observed in D50L plants.

**Figure 4:**
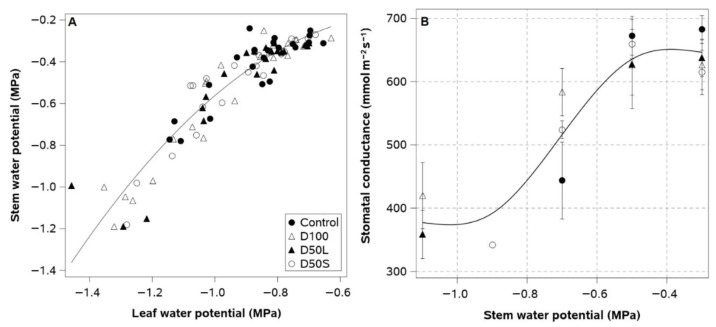
(**A**) Quadratic regression between midday values (12:00–15:30 h) of leaf and stem water potential (R^2^ = 0.83; *p*-value < 0.0001) and (**B**) B-spline fitting for the relationship between midpoints of stem water potential reflecting water stress severity levels and stomatal conductance measured from anthesis to early leaf senescence (November–April) of ‘Soreli’ kiwifruit vines subjected to four irrigation treatments (Control: 100% ET_c_, D50S: 50% ET_c_ for three weeks before harvest, D50L: 50% ET_c_ for five weeks before harvest, and D100: 0% ET_c_ for three weeks before harvest) in 2017 and 2018 seasons. Error bars represent ±1 se.

Leaf water potential exhibited a quadratic relationship with stem water potential and stomatal conductance (R^2^ = 0.83, *p* < 0.0001; Figure 4A). A small reduction of 0.1 MPa in stem water potential, from −0.3 to −0.4 MPa, was associated with a larger reduction of 0.3 MPa in leaf water potential, from −0.5 to −0.8 MPa. A sigmoid relationship was found between stem water potential and stomatal conductance throughout the season (Figure 4B). No change in stomatal conductance was observed within the SWP range of −0.5 to −0.3 MPa. For SWP between −0.8 and −0.5 MPa, stomatal conductance decreased linearly by 40%, reaching a minimum of 400 mmol m^−2^ s^−1^ when SWP was −1.0 MPa.

**Figure 5:**
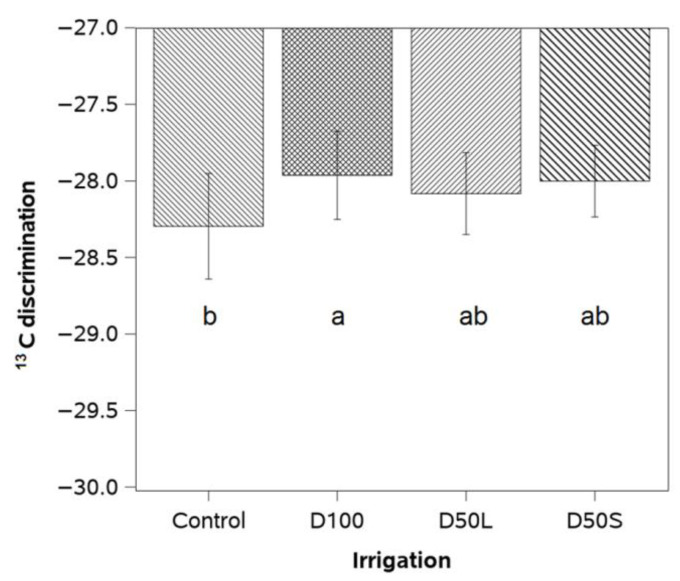
^13^C discrimination measured at harvest time (1st week of March 2018) in mature leaves of ‘Soreli’ kiwifruit vines subjected to four irrigation treatments (Control: 100% ET_c_, D50S: 50% ET_c_ for three weeks before harvest, D50L: 50% ET_c_ for five weeks before harvest, and D100: 0% ET_c_ for three weeks before harvest). Different letters indicate significant differences among treatments at *p* < 0.05 by LSD test. Error bars represent ±1 se.

There was no significant interaction between irrigation and season for ^13^C discrimination. Therefore, data from both years were pooled and presented. Leaves from D100 plants exhibited less negative values of ^13^C discrimination (~1.5%) than the Control, while values from D50S and D50L plants were similar to Control (Figure 5).

**Figure 6:**
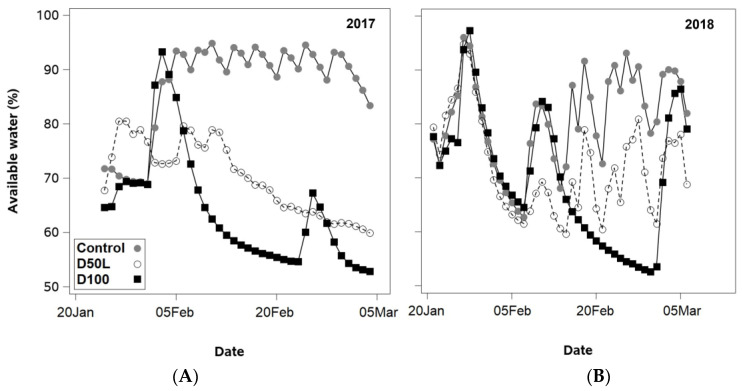
Daily values of percentage of available water averaged over two depths (−30 and −60 cm) from the onset of fruit maturation to harvest (January to March) of ‘Soreli’ kiwifruit vines subjected to three irrigation treatments (Control: 100% ET_c_, D50L: 50% ET_c_ for five weeks before harvest, and D100: 0% ET_c_ for three weeks before harvest) in (**A**) 2017 and (**B**) 2018 seasons.

In both years, the D100 treatment had the lowest soil water content, reaching nearly 53% of available water (see Figure 6A,B). In the first year, the control treatment ranged from 85% to 95% of available water. In the second year, there was greater variation in the control treatment’s SWC, with available water ranging from 70% to 95%. During the first year, the D50L treatment exhibited a similar SWC pattern to the control until the third week of February, when SWC decreased progressively. In fact, the minimum SWC of 60% of available water was only reached once, at the end of the season. In the second year, the D50L treatment exhibited a SWC change pattern like the control treatment, reaching the seasonal minimum of 60% of available water several times.

### 3.4. Vegetative and Reproductive Growth

**Figure 7:**
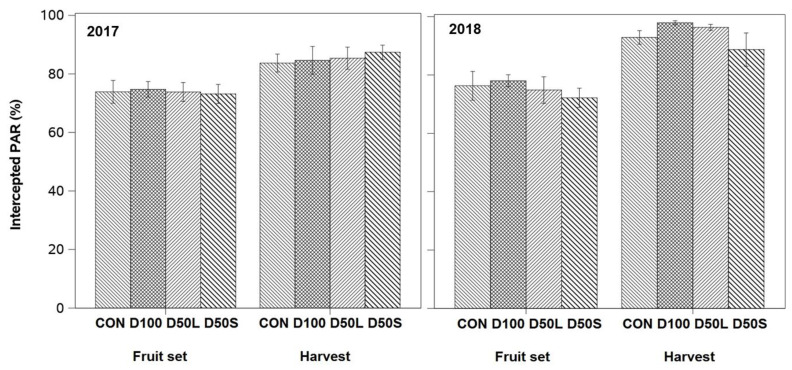
Percentage of Photosynthetically Active Radiation intercepted (% PAR) by the canopy of ‘Soreli’ kiwifruit vines during fruit set and harvest, subjected to four irrigation treatments in the 2017 and 2018 seasons. Treatments included CON (Control: 100% ET_c_), D50L (50% ET_c_ for five weeks before harvest), D50S (50% ET_c_ for three weeks before harvest), and D100 (0% ET_c_ for three weeks before harvest). Error bars represent ±1 se.

There were no differences in vegetative growth among irrigation treatments, measured as shoot length and number of leaves. In general, all treatments intercepted between 70% and 75% of PAR at fruit set each season (see Figure 7). At harvest, all plants intercepted at least 80% of PAR at harvest, which was increased by 10% in the second season.

**Figure 8:**
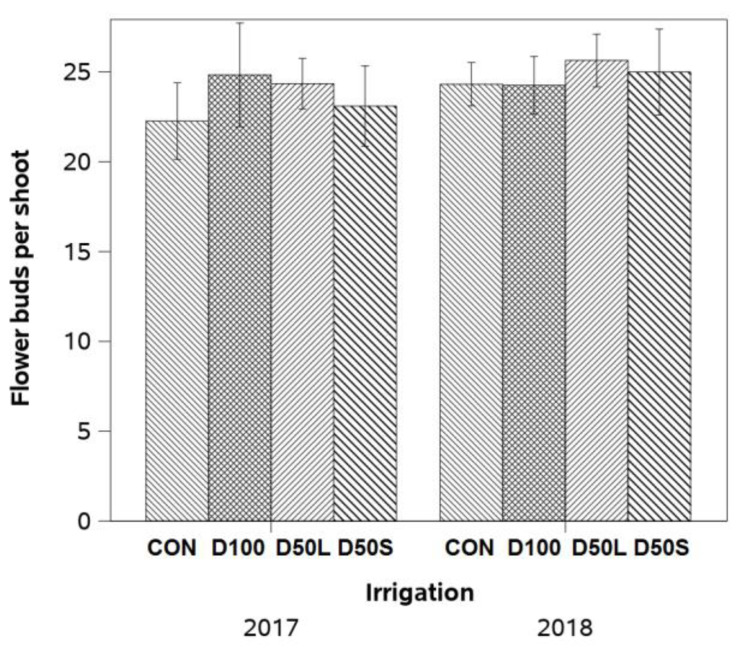
Number of flower buds per shoot measured at 50% anthesis (1st and 2nd week of November) in the following season on ‘Soreli’ kiwifruit vines subjected to four irrigation treatments in the 2017 and 2018 seasons. Treatments included CON (Control: 100% ET_c_), D50S (50% ET_c_ for three weeks before harvest), D50L (50% ET_c_ for five weeks before harvest), and D100 (0% ET_c_ for three weeks before harvest). Note: “CON” refers to the Control treatment and was abbreviated in the figure due to space limitations. Different letters indicate significant differences among treatments at *p* < 0.05 according to LSD test. Error bars represent ±1 se.

The number of flower buds per shoot was not affected by irrigation treatments, ranging in both years between 22 and 25 flower buds per shoot (see Figure 8).

**Figure 9:**
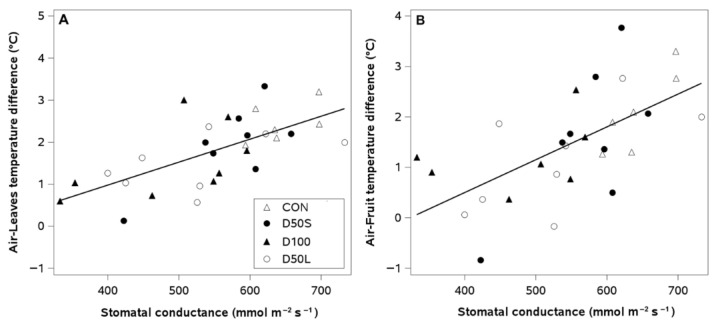
Linear regressions between the stomatal conductance and the temperature difference between the ambient air and (**A**) leaves (R^2^: 0.44; y = −1.21 + 0.0055x; *p* < 0.0001); y = and (**B**) fruits (R^2^:0.38; y = −2.1 + 0.0065x; *p* < 0.0003) from dates when deficit irrigation treatments changed stem and leaf water potentials in the second season (28 February and 16 March 2018) in ‘Soreli’ kiwifruit vines subjected to four irrigation treatments (Control: +100% ET_c_, D50S: 50% ET_c_ for three weeks before harvest, D50L: 50% ET_c_ for five weeks before harvest, and D100: 0% ET_c_ for three weeks before harvest); N = 30. Error bars represent ±1 se.

Regression analyses showed positive linear relationships between stomatal conductance and the temperature differential between fruits, leaves, and ambient air across all irrigation treatments (Figure 9A,B). Higher stomatal conductance values were associated with an increase in the air–leaf temperature difference close to 2.5 °C (Figure 9A). Similarly, the air–fruit temperature difference increased with higher stomatal conductance, reaching a maximum temperature difference near 3 °C within the same range of stomatal conductance (Figure 9B).

### 3.5. Fruit Quality at Harvest

In the first season, D100 had a higher content of soluble solids (~1.0 ºBrix) than the Control at harvest time (Table 3). In the second season, fruit was harvested ten days later and at a higher soluble concentration than in the first season (between 1.0 and 1.5 °Brix). In the second season, soluble solids concentration was significantly higher in D50S and D100 treatments. In the first season, irrigation had no effect on fruit firmness. However, in the second season, fruit from D50L and D50S was 6% less firm than the Control. The percentage of dry matter was 1% higher in fruit from D100 plants than in Control plants during the first season, but no irrigation effect on dry matter was detected during the second year. Polar and equatorial diameters were similar among treatments in the first season, but the equatorial diameter of fruits from D50S was ~1.0 cm lower than those from D50L. Whereas deficit irrigation treatments had no effect on the weight of the whole fruit, seed weight was higher by 10% in all water deficit treatments in the second year. The color of the fruit was impacted by the deficit irrigation, both in the pulp and the skin. However, this impact was not uniform from year to year. The skin in fruit from D100 plants reached slightly lower hue values than the control in the first season, but not in the second season. The pulp hue was lower in D100 than in the Control in the second season.

After 60 days of cold storage, the fruits exhibited no impact of irrigation on the concentration of soluble solids or the fresh weight in both seasons. In the first year, D100 exhibited firmness values of fruit pulp and columella that were 54% and 154% higher, respectively, than the average of the remaining treatments. In the second year, the D50L was the only treatment that showed a lower pulp firmness than the Control (see Table 4).

### 3.6. Sensory Evaluations

**Figure 10:**
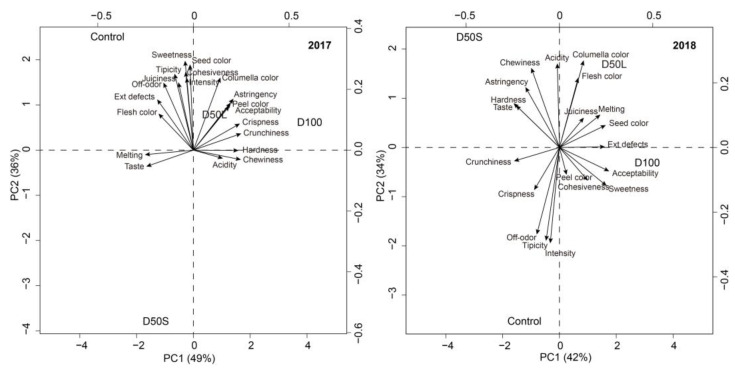
Principal component analysis (PCA) biplots in ‘Soreli’ kiwifruit vines subjected to four irrigation treatments (Control: 100% ET_c_, D50S: 50% ET_c_ for three weeks before harvest, D50L: 50% ET_c_ for five weeks before harvest, and D100: 0% ET_c_ for three weeks before harvest) after 60 days of storage at 0 °C during 2017 and 2018 seasons.

The PCA was used to determine the source of variation in the kiwifruit data of sensory parameters after 60 days of storage (See Figure 10). For the year 2017, PC1 explained 49% of the variation and 36% for PC2, whereas in 2018, it was 42 and 34% for PC1 and PC2, respectively. In the first year, the parameters taste, melting, hardness, acidity, and chewiness were strongly influencing PC1, and sweetness, seed color, cohesiveness, and aroma intensity were influencing PC2. In the second year, this behavior changed, showing that crunchiness and external defects are influencing PC1, whereas acidity, aroma typicity, aroma intensity, and off-odor are heavily loaded on component 2. Interestingly, for both years, acceptability correlated with peel color, while off-odor correlated with typicity and intensity of aroma. Notably, for 2017, the quality parameters (variable loadings) were grouped together, but in 2018, there was no clear pattern. The sample scores (irrigation treatments) were somewhat scattered in the PCA biplot; however, the treatments D50L and D100 were closer between them than the others on both years. These two treatments were more closely associated with positive quality parameters (e.g., acceptability).

## 4. Discussion

The implementation of deficit irrigation during the final three weeks of fruit growth and development in the kiwifruit cultivar ‘Soreli’ exhibited an enhancement in water savings, ranging from 10% to 20%. This water saving was not accompanied by adverse alterations in vegetative and reproductive growth. The observed 9% reduction in water application during the second season was accompanied by a 10% increase in PPFD interception, suggesting that the reduction in applied water created optimal conditions for sustaining vegetative growth in kiwifruit. In the present study, the amount of irrigation water in the first season was 40% higher than that recommended in previous studies for pergola-trained kiwifruit vines [5]. In this context, the reduction in water applied to an orchard with abundant irrigation may have reduced hypoxic soil conditions, which can enhance vegetative growth [29].

Before the implementation of deficit irrigation treatments, midday SWP and LWP measurements in all plants remained within a range indicative of well-irrigated conditions for kiwifruit (LWP values between −0.9 and −0.7 MPa) during each season [30]. A reduction in plant water status was measured a couple of weeks before the application of irrigation treatments in 2018, and all plants reached a moderate level of water stress (SWP ± −0.8 and LWP ± −1.0 MPa) when the atmospheric evaporative demand was the highest of the season (mid-January). This decline in plant water status was caused by a one-week reduction in water supply to the entire orchard due to an unexpected failure of the irrigation system. The occurrence of moderate water stress under high evaporative demand may have permanently compromised the xylem hydraulic conductivity of the entire orchard. Kiwifruit has been reported to be vulnerable to cavitation at SWP values near −1.0 MPa and to exhibit ineffective mechanisms to repair xylem embolism [8]. However, once irrigation was resumed, SWP and LWP in all plants recovered to well-watered conditions. This suggests that a moderate water stress of −0.8 MPa did not cause lasting damage to the vascular system of ‘Soreli’ plants. The application of deficit irrigation led to a notable decrease in plant water status near harvest time each year. However, the irrigation impact on SWP and LWP of vines irrigated at 50% of the control level was more evident in the second season. This is likely attributable to the combined effect of increased evaporative demand from January to March and the reduced water application in 2018. In contrast, plants subjected to the most severe water restriction (D100) reached a severe level of water stress (LWP ± −1.3 MPa) one week before commercial harvest operations in both seasons. The D100 treatment was the only one to achieve a progressive and consistent 50% reduction in available water. The remaining deficit irrigation treatments exhibited transient periods of soil drying and rewetting, reaching a minimum available soil water of 60%. The variations in soil drying patterns among deficit irrigation treatments may have been the cause of the differences in stomatal closure in response to similar levels of water stress in 2018. These different stomatal responses explain why D50L and D100 plants reached the same water stress severity at the end of the season, but D100 plants exhibited a 15% higher reduction in stomatal conductance of leaves than D50L plants. These results suggest that the dryness level of the soil likely served as a more influential driver of stomatal closure than the water status of the plants [31].

The quadratic regression of leaf and stem water potential observed in the present study was not consistent with previous findings in other fruit crops, in which a linear relationship between both variables has been reported. Generally, leaf water potential measurements indicate the severity of water stress in actively transpiring foliage, while stem water potential is a reliable indicator of water stress in the entire plant [24]. Therefore, under high plant water status, the lack of change in SWP over a 0.2 MPa reduction in LWP indicates that the onset of dehydration in exposed leaves is uncoupled from the rest of the plant. This suggests that initial departures from optimal leaf water status may depend more on atmospheric water demand than on soil desiccation. Thus, measuring stem rather than leaf water potential may be more convenient in maintaining kiwifruit plants under optimal water conditions. The high dehydration rate of leaves from well-watered kiwifruit plants is consistent with the lack of stomatal closure when SWP was above −0.5 MPa, which clearly shows no limitation in leaf transpiration under optimal water conditions. Once stomatal closure began (SWP < −0.5 MPa), reductions in SWP and LWP became directly proportional, suggesting that both plant-based measurements should be similarly accurate as indicators of water stress in deficit-irrigated kiwifruit plants.

The lowest SWP registered in this experiment was not low enough to reduce stomatal conductance to values that may limit transpiration in other fruit crops. For instance, in the present study, moderately water-stressed plants (SWP~−0.8 MPa) exhibited stomatal conductance levels of around 400 mmol m^−2^ s^−1^, which is considered high in apple, table grapevine, and sweet cherry. It has recently been suggested that a SWP value of −0.8 MPa may represent a threshold below which stomatal conductance began to decrease significantly in a 9-year-old mature commercial kiwifruit (*Actinidia chinensis* var. chinensis, cv. Zesy002) [31]. Our findings demonstrate that genotype differences can influence the stomatal response of kiwifruit plants to water stress. This suggests that certain cultivars, such as ‘Soreli,’ exhibit increased sensitivity to drought and cavitation. This sensitivity may be attributed to weaker stomatal control in response to decreased plant water status when compared to other kiwifruit cultivars grown under Mediterranean climate conditions. In almonds, Spinelli et al. [32] reported that inducing mild to moderate water stress caused as much as a 50% decrease in stomatal conductance, but no change in evapotranspiration. This suggests that, under high evaporative demand, water stress-induced reductions in stomatal conductance may be relatively ineffective in limiting plant dehydration.

In general, plants subjected to deficit irrigation showed less negative δ^13^C values, but only D100 plants exhibited a significant difference compared to the Control. This finding is consistent with the observation that D100 induced the most pronounced stomatal closure among the irrigation treatments. In C3 plants, the partial closure of stomata in response to water stress reduces CO_2_ diffusion in leaves and increases the use of ^13^C by the Rubisco enzyme [7]. Thus, in our study, D100 plants tended to be enriched in ^13^C as the severity of water stress increased to –1.3 MPa, and stomatal conductance decreased to 30% of the control treatment. In a recent study, Calderón-Orellana et al. [7] reported that the fruit from deficit and well-irrigated kiwifruit vines (cv. Hayward) exhibited similar δ^13^C ratios at harvest time, regardless of significant differences in midday leaf water potential during the season. These results suggest that the role of deficit irrigation in δ13C signatures is as variable as the stomatal response to water stress among cultivars and irrigation practices. From a practical perspective, our findings demonstrated that maintaining kiwifruit plants cv. ‘Soreli’ under moderate water stress by continuously dehydrating the soil resulted in slight improvements in iWUE [33].

In the present study, fruit was harvested when the concentration of soluble solids was higher than 6.5 °Brix, but the commercial target to collect the fruit was not the same each year. In the first season, harvest operations started in all irrigation plots when fruit from control plants reached 6.4 °Brix, while in the second season, harvest started at 9.4 ºBrix. The objective of collecting fruit at higher maturity in 2018 was to decrease the flesh softening after long periods of cold storage [34] and respond to recommendations from the Chilean kiwifruit industry. When fruit was harvested at lower maturity in the first season, D100 increased the soluble solids concentration, but had no effect on firmness. These results are consistent with previous studies of both green- and yellow-fleshed cultivars, in which deficit irrigation increased sugar content and maintained or increased fruit firmness [10,35]. Conversely, when the harvest date was postponed and fruit was picked at a higher maturity level the following year, D50S and D100 exhibited higher °Brix in comparison to Control. However, the impact of deficit irrigation on fruit firmness differed in the second season. While D50S exhibited softer fruit than Control, D100 reached the same level of firmness as the Control treatment each year. Kiwifruit is a climacteric fruit, in which the rates of ripening and softening are linked to higher fruit respiration, ethylene emission and starch degradation [36]. In kiwifruit, the onset of starch degradation occurs between 6.5 and 8.0 °Brix [37] and coincides with a considerable reduction in fruit firmness [38]. This implies that fruit from all irrigation treatments, but especially from severely water-stressed plants, probably transitioned from maturation to the ripening stage several days before the harvest date in 2018. This might suggest that, in D50S plants, the rise in soluble solids concentration at harvest was due to increased rates of starch degradation into soluble sugars, such as fructose, glucose, and sucrose, inducing higher fruit softening. Conversely, in D100 plants, the increase in °Brix at harvest, regardless of the maturity level, may indicate higher rates of soluble sugars allocation to fruits rather than higher rates of starch degradation. The results of the present study demonstrated that fruit from plants that reached SWP values below −0.8 MPa at the end of the growing season tended to exhibit an increase in the concentration of soluble solids. In apple (*Malus domestica* Borkh.), another climacteric fruit crop, mild water stress enhanced the activity of enzymes like sucrose synthase and acid invertase, which are crucial for converting sucrose into fructose and glucose. These water stress-induced changes in enzymatic activity were associated with higher sugar concentrations in fruits, suggesting that variation in the severity and duration of water stress has a differential impact on key enzymes involved in carbohydrate dynamics in fruits [39]. In fact, the inconsistent impact of deficit irrigation on fruit firmness and softening confirms the importance of variation in the severity and duration of water stress in determining fruit attributes. For instance, in the first season, only D100 plants reached SWP values below −0.8 MPa. This explains why only this irrigation treatment showed higher quality attributes at harvest. In the second season, D50S, D50L, and D100 plants surpassed that level of water stress, but the impact on fruit quality at harvest was not uniform across the various deficit irrigation treatments. Despite the lack of significant differences in one out of two years, peel and flesh from D100 plants were consistently less green and more yellow than the Control, as hue values were slightly lower. The color of the peel and flesh in kiwifruit is related to their chlorophyll concentration [40]; therefore, D100 plants may have triggered a decrease in chlorophyll concentration, suggesting oxidative damage and chlorophyll degradation. Another possibility that could explain these changes in fruit color may be related to changes in the temperature of the fruit of deficit-irrigated plants. The greater severity of water stress at the end of the season caused a nearly 50% reduction in stomatal conductance, which directly affected fruit temperature. This was evident in the regression analysis of leaf stomatal conductance and the temperature differential between the air and fruit during the second season. Water-stressed plants, whose conductance decreased by nearly 50%, had fruit temperatures almost identical to air temperatures. In kiwifruit, fruit transpiration decreases considerably at the end of the season, by about tenfold between fruit set and near harvest [41]. Therefore, fruits near harvest that transpire less lose their ability to dissipate heat through evaporative cooling. In kiwifruit plants, whose fruits are mostly shaded by the dense canopy, fruit temperature is largely dependent on the air temperature. If a plant reduces its stomatal conductance by 50%, the energy that would have been used to evaporate water remains as sensible heat, raising the temperature of leaves and fruits. Thus, higher fruit temperatures lead to a faster rate of fruit maturity and changes in fruit color. While these changes in fruit color could be a problem for green-fleshed kiwifruit cultivars, it is a positive quality attribute for yellow-fleshed kiwifruit, such as ‘Soreli’. Indeed, the hue values of pulp from D100 plants at harvest in the second season were near the average value for ‘Soreli’ fruits picked at a similar concentration of soluble solids in Central Chile [42].

After 60 days of cold storage, fruit analyses indicated that D100 plants produced fruits that were substantially firmer than the remaining treatments in the first season. However, these results were not replicated in the second season, when the harvest date was postponed by several days, resulting in fruit that was already in the ripening stage. In that season, fruit from D50L exhibited lower flesh firmness compared to the Control. These results are not consistent with observations at harvest time, when D50S was the only treatment that showed a significantly lower flesh firmness than the Control. These findings indicate that fruit from D100 maintained its quality attributes even after 60 days of cold storage, confirming that the higher concentration of soluble solids observed at harvest in the second season was not linked to increased starch degradation. In addition, the PCA indicates that the sensory quality attributes of kiwifruit were influenced by seasonal variation and irrigation treatments. While 2017 exhibited a cohesive grouping of quality traits, 2018 demonstrated a more dispersed pattern, indicating potential environmental or agronomic variability. This is not surprising, as in this season there was more variation in plant water status values and soil water content. Despite these differences, treatment D100 consistently showed favorable sensory attributes, particularly acceptability and peel color. These results highlight the key role of irrigation strategies in preserving fruit quality during storage.

## 5. Conclusions

The findings of the present study indicated that the most effective irrigation strategy was to completely cease irrigation three weeks prior to harvest. This approach resulted in water savings of up to 20%, without compromising yield or fruit quality.

From a physiological perspective, the most severe deficit irrigation treatment enhanced soil dehydration and stomatal regulation, leading to improved water use efficiency and fruit quality, even after extended cold storage. However, the impact of deficit irrigation on fruit firmness and postharvest quality varies depending on harvest maturity. Early harvests preserve firmness, while delayed harvests accelerate ripening and softening in deficit-irrigated plants.

Most of the prior deficit irrigation research in kiwifruit has focused on green-fleshed cultivars, such as ‘Hayward’. This study specifically investigates the yellow-fleshed ‘Soreli’, which has different physiological traits and market relevance, making the findings cultivar-specific and novel.

The implementation of regulated deficit irrigation (RDI) in an emerging, yellow-fleshed cultivar like ‘Soreli’, which is gaining popularity in Mediterranean regions, is particularly relevant in the context of climate change. This study confirmed that RDI is a viable, adaptive irrigation strategy for kiwifruit production in the face of increasing water scarcity.

## Figures and Tables

**Table 1:** Applied water, yield, water productivity, and difference in water productivity in ‘Soreli’ kiwifruit vines from three irrigation treatments Control: 100% ET_c_, D50S: 50% ET_c_ for three weeks before harvest, D50L: 50% ET_c_ for five weeks before harvest, and D100: 0% ET_c_ for three weeks before harvest in 2017 and 2018 seasons.

	Applied Water (m^3^ ha^−1^)	Yield (kg ha^−1^)	Water Productivity (kg m^−3^)	Difference in Water Productivity (%)
2017				
Control	14,774 a	27,133	1.84	0.0%
D50S	13,875 b	27,791	2.00	9.1%
D50L	12,924 b	28,410	2.20	19.7%
D100	11,766 c	25,766	2.19	19.2%
2018				
Control	13,520 a	27,163	2.01	0.0%
D50S	12,697 b	27,788	2.19	8.9%
D50L	12,594 b	28,413	2.26	12.3%
D100	12,309 c	25,763	2.09	4.2%

Within-column means followed by different letters indicate significant differences among treatments at *p* < 0.05 by LSD test (n = 4).

**Table 2:** Stomatal conductance of leaves measured at midday (12:00–15:30 h) in ‘Soreli’ kiwifruit vines from three irrigation treatments, Control: 100% ET_c_, D50S: 50% ET_c_ for three weeks before harvest, D50L: 50% ET_c_ for five weeks before harvest, and D100: 0% ET_c_ for three weeks before harvest in 2018 season.

Date	Irrigation Treatments			
	Control	D50S	D50L	D100
	Stomatal Conductance (mmol m^−2^ s^−1^)			
23-Nov-17	715.7	704.7	699.4	678.2
23-Dec-17	734.9	697.4	788.3	708.9
9-Jan-18	567.3	512.6	598.9	612.3
2-Feb-18	767.2	735.8	714.4	720.7
28-Feb-18	667.6 a	602.4 a	583 a	426.4 b
16-Mar-18	613.3 a	540.7 ab	472.9 b	555.3 a

Within-column means followed by different letters indicate significant differences among treatments at *p* < 0.05 by LSD test (n = 4).

**Table 3:** Fruit quality parameters in ‘Soreli’ kiwifruit vines from four irrigation treatments (Control: 100% ET_c_, D50S: 50% ET_c_ for three weeks and D50L: 50% ET_c_ for five weeks before harvest, and D100: 0% ET_c_ for three weeks before harvest) in 2017 and 2018 seasons.

	Treatments			
	Control	D50S	D50L	D100
2017				
Soluble solids Concentration (°Brix)	6.84 b	7.30 b	7.47 b	9.31 a
Firmness (lb)	13.6	13.6	13.0	13.2
Dry matter (%)	15.9 b	16.9 a	16.3 ab	16.7 ab
Fresh weight (g)	122.3	118.7	125.7	117.4
Equatorial diameter (mm)	52.4	52.2	52.9	51.7
Polar diameter (mm)	71.4	70.2	71.6	70.2
Skin hue	90.7 a	88.5 ab	89.3 ab	87.2 b
Pulp hue	100.8	100.0	99.8	99.4
2018				
Soluble solids Concentration (°Brix)	9.43 b	10.5 a	10.3 ab	10.6 a
Firmness (lb)	12.37 a	11.65 b	11.66 b	11.75 ab
Dry matter (%)	16.4	17.07	16.9	16.9
Fresh weight (g)	112.8	111.9	114.9	110.6
Equatorial diameter (mm)	52.9 ab	52.0 a	53.2 b	52.8 ab
Polar diameter (mm)	65.5	65.9	65.0	65.3
Seed weight (g)	3.43 b	3.75 a	3.85 a	3.91 a
Peel hue	86.9	85.8	85.6	85.2
Flesh hue	104.1 a	102.2 ab	100.6 b	100.7 b

Within-column means followed by different letters indicate significant differences among treatments at *p* < 0.05 by LSD test (n = 4).

**Table 4:** Fruit quality parameters in ‘Soreli’ kiwifruit vines from four irrigation treatments (Control: 100% ET_c_, D50S: 50% ET_c_ for three weeks before harvest, D50L: 50% ET_c_ for five weeks before harvest, and D100: 0% ET_c_ for three weeks before harvest) in 2017 and 2018 seasons.

	Treatments			
	Control	D50S	D50L	D100
2017				
Soluble solids concentration (°Brix)	13.9	14.1	13.8	14.1
Pulp firmness (lb)	4.83 b	5.47 b	5.81 b	8.11 a
Columella firmness (lb)	4.48 b	5.71 b	6.52 b	14.2 a
Fresh weight (g)	111.2	107	110.1	101.8
2018				
Soluble solids concentration (°Brix)	13.4	13.8	13.6	13.8
Pulp firmness (lb)	4.07 a	3.65 ab	2.31 b	2.70 ab
Columella firmness (lb)	9.55	7.07	8.37	8.42
Fresh weight (g)	114.9	105.3	111.5	114.4

Within-column means followed by different letters indicate significant differences among treatments at *p* < 0.05 by LSD test (n = 4).

## Data Availability

The original contributions presented in this study are included in the article. Further inquiries can be directed to the corresponding author.

## Supplementary Materials

The following supporting information can be downloaded at: https://www.mdpi.com/article/10.3390/plants14182843/s1, Figure S1. Canopy of kiwifruit plants subjected to treatments by irrigation strategies applied. (A) Control: plants irrigated at 100% of ETc throughout the growing season; (B) D50L: plants irrigated as the control from bud break until five weeks before commercial harvest, then at 50% of irrigation until fruit harvest; (C) D50S: plants irrigated as the control from sprouting until three weeks before commercial harvest; and (D) D100: plants irrigated as the control from sprouting until three weeks before commercial harvest.
