# Supplementary material for: Late Water Deficits Improve Intrinsic Water Use Efficiency, Fruit Maturity, and Acceptability in Yellow-Fleshed Kiwifruit cv. Soreli
Source: Plants (Basel). 2025 Sep 12;14(18):2843. doi: 10.3390/plants14182843 (PMC12473459; doi:10.3390/plants14182843)
Supplement: Supplementary file 1 [file plants-14-02843-s001.zip › plants-3820378-supplementary.pdf]

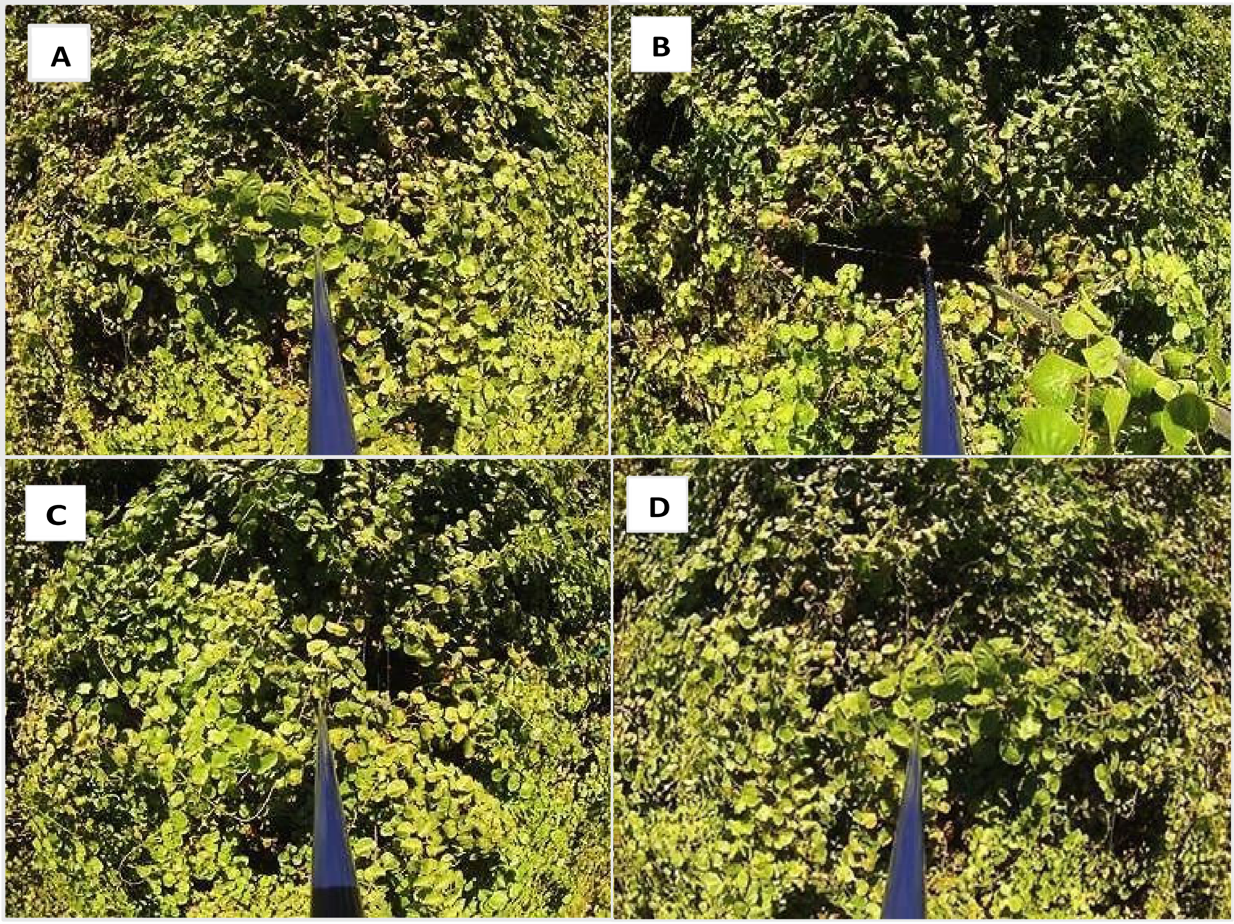

Supplementary Figure S1. Canopy of kiwifruit plants subjected to treatments by irrigation strategies applied. **(A) Control**: plants irrigated at 100% of ETc throughout the growing season; **(B) D50L**: plants irrigated as the control from bud break until five weeks before commercial harvest, then at 50% of irrigation until fruit harvest; **(C) D50S**: plants irrigated as the control from sprouting until three weeks before commercial harvest; and **(D) D100**: plants irrigated as the control from sprouting until three weeks before commercial harvest.
